# Supplementary material for: Modeling time-varying brain networks with a self-tuning optimized Kalman filter
Source: PLoS Comput Biol. 2020 Aug 17;16(8):e1007566. doi: 10.1371/journal.pcbi.1007566 (PMC7451990; doi:10.1371/journal.pcbi.1007566)
Supplement: Attachment — Submitted filename: review_v2.docx [file pcbi.1007566.s001.docx]

In this paper, Pascucci et al. have suggested a self-tuning Kalman filter that can track the changes in the data and tune the Kalman filter free parameter. They have designed some simulations to showcase their method benefits compared to Kalman filter with fix c. In addition, they have used their methods to explore the dynamics of two separate real world dataset. All in all, I find this paper very interesting and I think changing the tuning parameter of the Kalman filter is very useful, but I think some additional simulations are needed for readers to understand the advantage and disadvantages of this method (I do believe the advantages outweigh the disadvantages).

Major comments:

1. My biggest issue with the paper is with the simulations. I think additional simulations are needed to showcase additional aspects of STOK.
   a. What will happen if the changes in the AR coefficient is somewhat fast that don’t allow STOK to reach a stable tuning state? Essentially, I am asking if there is a limiting resolution to how fast STOK can tune (or if some variations are not seen by STOK).
   b. In the fist simulation, the AR coefficient start from very low values and c in STOK is low too. Then the changes in AR coefficient happen in the middle of the time series. What will happen if AR coefficient are high from start and changes before c is tuned completely. Can u show a situation like that? (I think you have simulated that as I see in the method section that the onset is randomized too).
   c. What would happen if the **change** in AR coefficient is not as prominent as what has been simulated? If coefficient change in smaller sizes can STCK tune c again? What is the lower bound in change in coefficients that STOCK can see? Please note that I am not asking how fast it changes (as in part a), I am asking the value of change is small.
   d. In Figure 1B, I see that change in c is only happen once when AR coefficient changes and then go back to low values. I was expecting that c start changing (go high) while the AR coefficient is high then go down when AR coefficient goes to zero again. Can the authors explain this behavior? And does it have anything to the initial value of c? I mean if you select c=0.5 as initial value for STOK will it go back to c=0.5 after the jump?
   e. Why have the authors chosen to run their simulations only 200 times? This is rather low for a simulation. Or why 30 realization as mentioned in line 823?
2. There are some inconsistencies with how the statistics are reported. For example, in line 159, 207, and 216 p<0.001 is stated to show significance. But in line 162, 211 p<0.01 is stated as significant. Then in line 192 p=0.009 is reported. And in the line 162 p>0.05 is stated as non-significance? This is a problem especially that all of these are for the simulation parts. Please unify all of the test (and how you report them)
3. On line 314 the authors mention that they have calculated correlation between PSD of different methods? How have they calculated correlation? 2D correlation or vectorized PSD and then calculate correlation.
4. What are the tuned c values for STOK for the real data experiments?

Minor comments:

1- I cannot access the GitHub page for the code.
2- I would suggest the authors to change some of the figures for better visibility and maximizing the information is convey to the readers (these are merely suggestions and authors can ignore if they like):
   a. Figure 1A the line for c=1 and for c=0.0001 are both very similar bluish colors.
   b. In Figure C and F some lines are overlaid on top of each other. This figure does not give readers any info about some of the lines as they are buried behind some other lines. Maybe include more transparency?
   c. Figure 4: white text on yellow is not really visible. Maybe change the color?
3- Some typos:
   a. In line 653 I think I_0_ should be I_N_
   b. Please use multiplication sign instead of the letter x, e.g. line 556
   c. Some references are not shown in the standard format, e.g line 226
