# Supplementary material for: Modeling time-varying brain networks with a self-tuning optimized Kalman filter
Source: PLoS Comput Biol. 2020 Aug 17;16(8):e1007566. doi: 10.1371/journal.pcbi.1007566 (PMC7451990; doi:10.1371/journal.pcbi.1007566)
Supplement: Attachment — Submitted filename: Pascucci_et_al_STOK_ReviewerResponse.docx [file pcbi.1007566.s002.docx]

# Response to Reviewers

Pascucci, Rubega & Plomp – PCOMPBIOL-D-19-02025

We would like to thank the Editor and the two reviewers for their positive evaluation of our work, as well as for the constructive comments and suggestions they provided. We have carefully considered each of the comments and revised our manuscript accordingly.

Below we provide a point-by-point response to each of the comments. The related changes in the manuscript are highlighted in red.

# Reviewer #1

In this paper, the authors present a modification of the Kalman filter with an adaptive tuning parameter, intended to adjust to the non-stationarities in the smoothness of the signal. The method is applied to both synthetic and EEG data, with convincing results.

In my opinion, this is a very solid methodological paper, although has no major neuroscience/biology contribution.

We thank the Reviewer for this positive evaluation and for recognizing the validity of our method and results.

I only have relatively minor comments:

- I can imagine the computational complexity could be an issue. The experimental data seems to be of small to moderate size (I could not find the duration of the data, please specify if missing). How would the method escalate to bigger data, both in terms of number of channels and number of time points?

We thank the Reviewer for this comment, which helps us to clarify some of the aspects of the proposed filter. In our ms, we presented simulations only up to 40 nodes. With such networks of moderate size, we showed that STOK outperforms the classical Kalman in retaining the strongest connections, due to the intrinsic regularization that promotes sparser solutions. This very same behaviour holds for larger networks: regularization ensures the least-squares estimation of AR coefficients even when the dimensionality (and potentially the redundancy and collinearity) of the problem increases. In this regard, therefore, computational complexity does not represent an issue. Increasing network size can increase the computational cost of inverting large matrices, but note that in STOK, matrix inversions are done using diagonalization to reduce round-off errors and speed the computation. Similarly, increasing the number of time points does not increase computational complexity, since recursive filters operate one time step at a time and thus, there is no limit for the duration of the data (the duration of the simulated data is 2 seconds, 200 Hz, as specified in line 800).

- "In its current form, the STOK filter is a multi-trial algorithm, leveraging regularities and correlations across trials under the assumption that multiple trials are coherent, temporally aligned realizations of the same process". Could the authors elaborate on whether the method could be applied, or adapted, to continuous data? Also, previous work has hinted to possible differences in durations of stimulus processing between trials [1,2]. Could the authors comment on how the method would behave to differences in such timing? I think it could be interesting to comment on this, even if briefly.

[1] Stokes MG, Spaak E. 2016. The importance of single-trial analy-ses in Cognitive Neuroscience. Trends Cogn Sci. 20:483–486

[2] Vidaurre D, Myers N. Stokes MG, Nobre AC, Woolrich MW 2019 Temporally Unconstrained Decoding Reveals Consistent but Time-Varying Stages of Stimulus Processing. Cerebral Cortex Volume 29: 863–874

This is indeed a very relevant point. Our algorithm, as well as the general Kalman filter, relies on the assumption that multiple trials are realizations of the same process, temporally aligned and consistent across trials. This, however, does not exclude the application to single trial or continuous data with proper adjustments, as already mentioned in the Discussion (line 496). Of course, in the case of multiple trials, a proper realignment and time-locking of the events of interest is recommended before STOK filtering when differences in the duration and onset of the process under investigation are to be expected. We have now expanded the discussion of this point and included the suggested references (lines 586-590).

- As a general comment, it feels to me that the Discussion is too lenghty and methods-oriented for a journal focused on Computational Biology. I would suggest to make it a bit less dense.

We thank the Reviewer for this suggestion, the Discussion section has been revised accordingly.

- Importantly, the two links that the authors provide for the code don't work. Given the type of contribution that this paper makes, and the complexity of the implementation, I believe it's fundamental that the authors provide code in good shape. I have responded No to the question of whether all data was made available for this reason.

We are sorry for this oversight, public github repository containing both the Matlab and Python version of our code can be found here:

<https://github.com/PscDavid/dynet_toolbox>

<https://github.com/joanrue/pydynet>

More minor:

- Please label the Figures more clearly. For instance, Fig 2F is confusing, what are exactly the two panels, and what is represented in the X-axis (given that the selected p is indicated in the title and the ground truth is 6)? Panels 3C don't have labels in the X-axis, etc.

Thank you, figures have been revised as suggested.

- Fig 3F, what happens with KF at 1000Hz? I wouldn't say these look like "similar dynamics". In 3D, KF at 1000Hz looks also strange. Isn't it at odds with the statement "Previous work has demonstrated that downsampling can have adverse effects on connectivity estimates"?

The reviewer is right in noticing the difference between KF results in figure 3F-D, where the Kalman filter presents a drastic decrease of tracking accuracy. This is indeed the consequence of keeping a high sampling rate which leads KF to overfitting, following noisy fluctuations more than the expected autoregressive patterns. The sentence has now been corrected to “Previous work has demonstrated that high sampling rates can have adverse effects…”.

# Reviewer #2

In this paper, Pascucci et al. have suggested a self-tuning Kalman filter that can track the changes in the data and tune the Kalman filter free parameter. They have designed some simulations to showcase their method benefits compared to Kalman filter with fix c. In addition, they have used their methods to explore the dynamics of two separate real world dataset. All in all, I find this paper very interesting and I think changing the tuning parameter of the Kalman filter is very useful, but I think some additional simulations are needed for readers to understand the advantage and disadvantages of this method (I do believe the advantages outweigh the disadvantages).

We thank the Reviewer for this positive assessment of our manuscript and for the important simulations suggested.

Major comments:

My biggest issue with the paper is with the simulations. I think additional simulations are needed to showcase additional aspects of STOK.

What will happen if the changes in the AR coefficient is somewhat fast that don’t allow STOK to reach a stable tuning state? Essentially, I am asking if there is a limiting resolution to how fast STOK can tune (or if some variations are not seen by STOK).

We thank the Reviewer for this comment, and we believe the suggested simulation is indeed critical for validating our filter. We have now included the results of a new simulation where networks are simulated with a single AR change that occurs with variable durations (ranging from 25 ms to 500 ms, figure 2D). In such a stringent test of tracking speed, we show good accuracy already for very fast AR changes (e.g. 25 ms), and that STOK clearly outperforms the classical Kalman filter.

In the first simulation, the AR coefficient start from very low values and c in STOK is low too. Then the changes in AR coefficient happen in the middle of the time series. What will happen if AR coefficient are high from start and changes before c is tuned completely. Can u show a situation like that? (I think you have simulated that as I see in the method section that the onset is randomized too).

In the main simulation (Figure 2), changes in AR coefficients were imposed to start after at least 300 ms from the trial onset (this information is now included in the revised ms). This is common practice in adaptive filtering to prevent artifacts due to the initial stages of the filter adaptation. Because adaptive filters (both the Kalman and the STOK) are initialized with small and uniform values (e.g., see Milde et al., 2010), it usually takes several time steps to calibrate from the model residuals and to reach proper tracking performance. Following the Reviewer’s question, we checked whether performance of STOK depends on the onset time of AR coefficients, using the same basic simulation as the one used for Figure 1 of our manuscript. As the following figure shows, tracking performance appears nearly identical when AR coefficients start at time zero, in the middle or at the end of a surrogate trial. As for real data, however, we would recommend using time segments which include a relatively long baseline period (e.g., before the process of interest) to prevent any edge artefact of the filter. This recommendation is now included in the paper (line 594).

What would happen if the **change** in AR coefficient is not as prominent as what has been simulated? If coefficient change in smaller sizes can STCK tune c again? What is the lower bound in change in coefficients that STOCK can see? Please note that I am not asking how fast it changes (as in part a), I am asking the value of change is small.

We thank the Reviewer for suggesting this important simulation. We have now included the results of a simulation manipulating the size of the AR changes in surrogate data, which shows how STOK performance is overall better compared to the Kalman filter, also when the AR coefficients change only slightly (Figure 2E).

In Figure 1B, I see that change in c is only happen once when AR coefficient changes and then go back to low values. I was expecting that c start changing (go high) while the AR coefficient is high then go down when AR coefficient goes to zero again. Can the authors explain this behavior? And does it have anything to the initial value of c? I mean if you select c=0.5 as initial value for STOK will it go back to c=0.5 after the jump?

In the current formulation, the constant *c* tracks changes — c increases when the current model does not account well for the current observation. This implies that if the structure of AR coefficients changes, then *c* increases, but when the AR coefficients remain stable (either high or low) then *c* decreases, since a stable process does not require any fast update of the filter. We hope this explanation clarifies the Reviewer’s concern.

Why have the authors chosen to run their simulations only 200 times? This is rather low for a simulation. Or why 30 realization as mentioned in line 823?

We designed our simulations in order to stay as close as possible to real data situations. For this reason, we choose a number of trials (200) that is acceptable and common in many event-related M/EEG studies with factorial designs and multiple conditions. Similarly, we used 30 realizations as 30 surrogate subjects in a typical event-related experiment. Nevertheless, confidence intervals in Figure 2 show that performance is rather robust with the chosen sample sizes.

There are some inconsistencies with how the statistics are reported. For example, in line 159, 207, and 216 p<0.001 is stated to show significance. But in line 162, 211 p<0.01 is stated as significant. Then in line 192 p=0.009 is reported. And in the line 162 p>0.05 is stated as non-significance? This is a problem especially that all of these are for the simulation parts. Please unify all of the test (and how you report them)

We thank the Reviewer for this comment. The reported p=0.009 in line 192 was indeed a typo. Since in most cases we report summary statistics for more than one comparison at time, we have now unified all p values according to the alpha criterion used as p<0.05 or p>0.05.

On line 314 the authors mention that they have calculated correlation between PSD of different methods? How have they calculated correlation? 2D correlation or vectorized PSD and then calculate correlation.

We used pairwise linear correlations between vectorized PSD, this information is now reported on line 380.

What are the tuned c values for STOK for the real data experiments?

The time-varying values of the self-tuning c for rat and human data are depicted in figures 3B and 4B, respectively, with their scale on the right-side y-axis.

Minor comments:

I cannot access the GitHub page for the code.

We are sorry for this oversight. We now provide the link for a public github repository containing both the Matlab and Python version of our code.

<https://github.com/PscDavid/dynet_toolbox>

<https://github.com/joanrue/pydynet>

I would suggest the authors to change some of the figures for better visibility and maximizing the information is convey to the readers (these are merely suggestions and authors can ignore if they like):

Figure 1A the line for c=1 and for c=0.0001 are both very similar bluish colors.

In Figure C and F some lines are overlaid on top of each other. This figure does not give readers any info about some of the lines as they are buried behind some other lines. Maybe include more transparency?

Figure 4: white text on yellow is not really visible. Maybe change the color?

Thank you for the suggestions, figures have been revised as suggested.

Some typos:

In line 653 I think I_0_ should be I_N_

Corrected as suggested.

Please use multiplication sign instead of the letter x, e.g. line 556

The letter x was used in line 556 to distinguish between multiplication and dimensions (as in line 643 where [d*p x p] stands for dimension 1: product of nodes and order; dimension 2: order).

Some references are not shown in the standard format, e.g line 226

Corrected as suggested.
